# SARS-CoV-2 Seroprevalence and Cross-Variant Antibody Neutralization in Cats, United Kingdom

**DOI:** 10.3201/eid2906.221755

**Published:** 2023-06

**Authors:** Grace B. Tyson, Sarah Jones, Nicola Logan, Michael McDonald, Leigh Marshall, Pablo R. Murcia, Brian J. Willett, William Weir, Margaret J. Hosie

**Affiliations:** Medical Research Council—University of Glasgow Centre for Virus Research, Glasgow, Scotland, UK (G.B. Tyson, S. Jones, N. Logan, P.R. Murcia, B.J. Willett, M.J. Hosie);; University of Glasgow, Glasgow (G.B. Tyson, S. Jones, M. McDonald, L. Marshall, W. Weir)

**Keywords:** COVID-19, SARS-CoV-2, severe acute respiratory syndrome coronavirus 2, viruses, respiratory infections, zoonoses, anthropozoonoses, One Health, cats, pseudotype virus neutralization assay, ELISA, humoral immunity, seroprevalence, United Kingdom

## Abstract

Anthropogenic transmission of SARS-CoV-2 to pet cats highlights the importance of monitoring felids for exposure to circulating variants. We tested cats in the United Kingdom for SARS-CoV-2 antibodies; seroprevalence peaked during September 2021–February 2022. The variant-specific response in cats trailed circulating variants in humans, indicating multiple human-to-cat transmissions over a prolonged period.

The World Organisation for Animal Health reported that 26 different animal species had been infected with SARS-CoV-2 by December 31, 2022; ≈30% (8/26) of the susceptible species are felids (*1*). Animal SARS-CoV-2 infections originating from anthropogenic transmission can lead to onward animal-to-animal transmission, as described previously in mink (*2*), hamsters (*3*), and white-tailed deer (*4*). There have also been reports of animal-to-human transmission of SARS-CoV-2 from farmed mink (*2*), pet hamsters (*5*), free-ranging white-tailed deer (*6*), and a pet cat (*7*).

It is unknown whether individual SARS-CoV-2 variants are more or less likely to be transmitted from humans to cats or whether infected cats are more or less likely to develop clinical signs. The aim of this study was to assess the seroprevalence of SARS-CoV-2 infection in cats during April 2020–February 2022 in the United Kingdom. We used a pseudotype-based neutralization assay (PVNA) to measure virus neutralizing antibody titers and a confirmatory ELISA that measured antibodies recognizing the receptor binding domain of the SARS-CoV-2 spike (S) protein. We measured neutralizing titers against a panel of viral pseudotypes based on a lentiviral (HIV) backbone and bearing the S proteins of the predominant circulating variants in the United Kingdom to investigate the specificity of the neutralizing response. The University of Glasgow Veterinary Ethics Committee granted approval for the study (EA27/20).

## The Study

**Figure 1:**
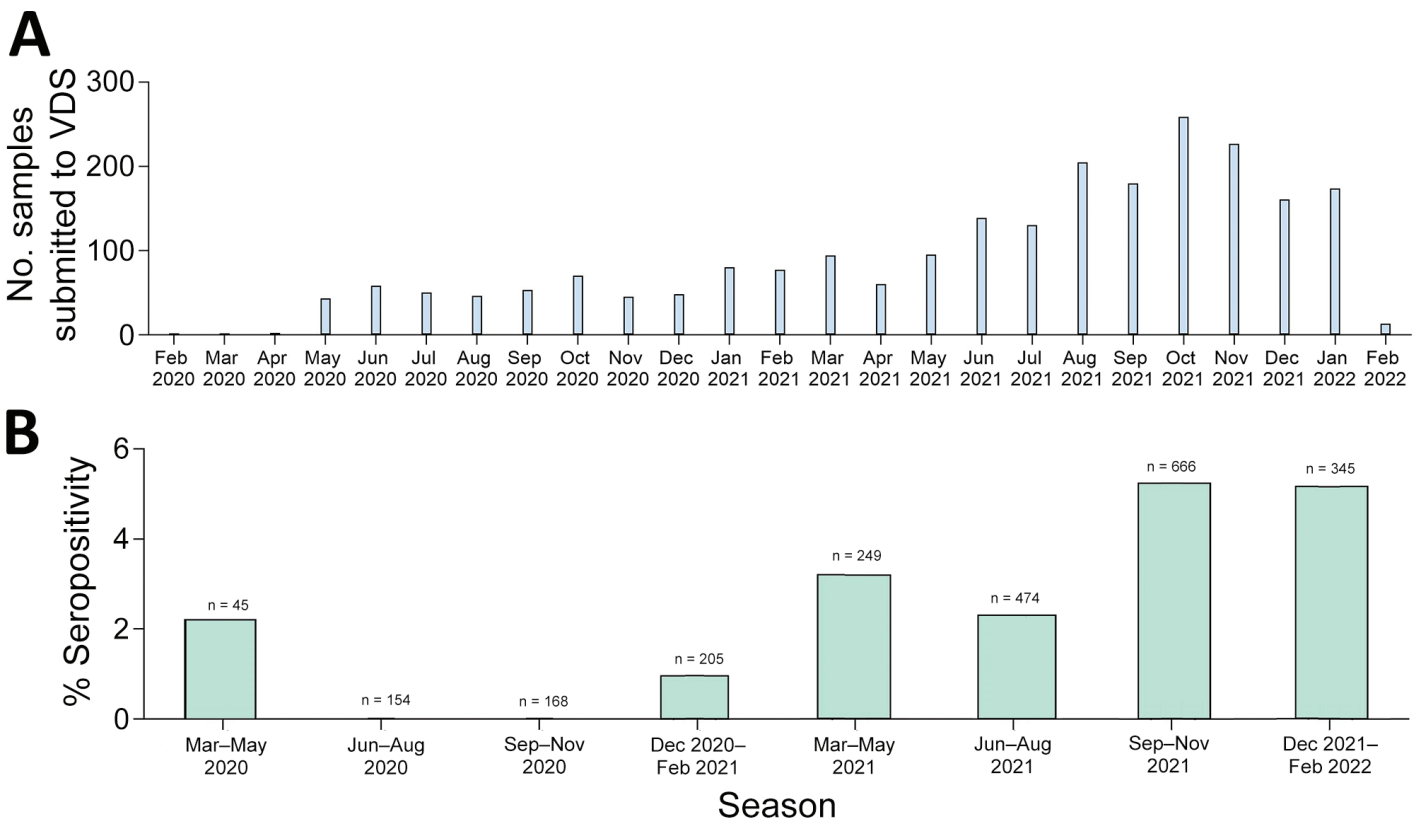
Seropositivity of samples included in analysis in study of SARS-CoV-2 seroprevalence and immunity in cats, United Kingdom, April 2020–February 2022. A) Number of samples tested per month. Overall seropositivity across all samples was 3.2% (75/2,309). B) Percentage seropositivity of samples per 3-month period and sample size for each period. VDS, University of Glasgow Veterinary Diagnostic Services laboratory.

We screened residual blood samples from 2,309 cats by using PVNA at a final dilution of 1:100; the samples were submitted to the University of Glasgow Veterinary Diagnostic Services laboratory (VDS) during April 2020–February 2022 (Figure 1, panel A). The samples represented a cohort that was broadly representative of the domestic cat population in the United Kingdom, including samples from 112 of the 126 UK postcode areas (Appendix 1 Figure 1), although the samples had an uneven distribution unrelated to the local human population density. Overrepresented areas included Blackpool, Glasgow, Edinburgh, and Cambridge. The PVNA used HIV (SARS-CoV-2) pseudotypes bearing S proteins of SARS-CoV-2 ancestral D614G (B.1), Alpha (B.1.1.7), Delta (B.1.617.2) or Omicron (BA.1). Samples submitted early in the pandemic were tested against ancestral D614G (B.1) only, whereas new variants were included as they emerged (Appendix 2). We estimated neutralization titers for positive samples by performing the PVNA with serially diluted samples. 

Our results showed that SARS-CoV-2 seroprevalence in UK cats increased over time (Figure 1, panel B). Overall, the seroprevalence during the study period was 3.2% (95% CI 2.56%–4.05%; 75/2,309). Seroprevalence was highest during September–November 2021 (5.3%, 95% CI 3.69%–7.23%; 35/666) and during December 2021–February 2022 (5.2%, 95% CI 3.09%–8.05%; 18/348).

**Figure 2:**
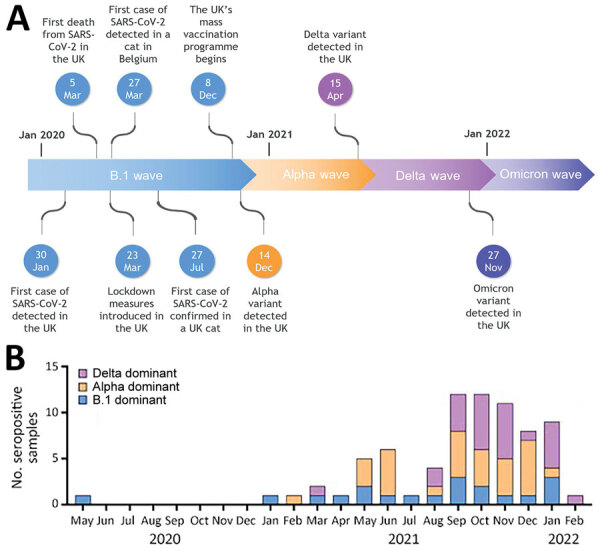
Dominant variant of seropositive samples by date in study of SARS-CoV-2 seroprevalence and immunity in cats, United Kingdom, April 2020–February 2022. A) Timeline of key events during the COVID-19 pandemic in the United Kingdom, including the emergence of major variants into the human population. B) Seropositive samples from cats, categorized by dominant variant and plotted by month. B.1 indicates ancestral/wild-type virus.

When we analyzed individual samples, we observed differences in variant-specific potencies among titers against the different SARS-CoV-2 variants: 17/75 (22.7%) samples were B.1 dominant (i.e., they possessed higher titers against B.1 than against other variants); 31/75 (41.3%) were Alpha dominant, and 27/75 (36%) were Delta dominant. On average, Delta-dominant samples displayed higher neutralization titers (mean 760) against their dominant pseudotype compared with Alpha-dominant (488; p = 0.06) or B.1-dominant (329; p = 0.02) samples (Appendix 1 Figure 2). Throughout the study period (April 2020–February 2022), no Omicron-dominant seropositive samples were identified; we anticipated this finding because only a small proportion of samples were collected after the Omicron variant emerged.

We observed an association between the dominant variant in cats and the timeline of variant emergence in the human population. Detection of new dominant variants in cats trailed detection of the variant in the humans; however, we detected dominant titers against extinct variants even after human cases had declined, possibly indicating long-lasting humoral immunity (Figure 2). We observed 3 distinct patterns of neutralization. B.1-dominant samples generally had slightly lower titers against the Alpha pseudotype than against B.1. Those samples also had significantly lower titers against both the Delta (p<0.0001) and Omicron (p<0.001) pseudotypes. Alpha-dominant samples showed slightly lower B.1 titers and markedly lower Delta and Omicron titers. Delta-dominant samples showed similar titers against the B.1, Alpha, and Omicron pseudotypes, all of which were significantly lower than their Delta titers (p<0.0001) (Appendix 1 Figure 3). 

The trends we observed for cats thought to have been infected with the B.1 variant are similar to the patterns of neutralization in humans reported previously (*8*); Wilhelm et al. showed that humans vaccinated with an ancestral strain–based vaccine develop lower neutralization titers against the Delta and Omicron variants than against B.1 or Alpha. Another study showed that cats experimentally inoculated with either the ancestral or the Delta variant became lethargic and pyrexic, whereas Omicron-inoculated cats did not develop any clinical signs and displayed lower levels of virus shedding, suggesting that the Omicron variant might be less pathogenic in cats as well as in humans (*9*).

Despite those distinct patterns of neutralization, the variant to which the animal was exposed can only be speculatively inferred through serologic testing in the absence of viral sequence data, even in cases in which the titer against the dominant variant is many times greater than the next highest titer. The 3 specific patterns of immunity we observed were similar to previous findings in humans (*10*). It is likely that both the antigenicity of the different variants’ S proteins and the viral load during the infection period influence the breadth and potency of variant-specific neutralization.

A greater proportion of purebred cats (31/720 [4.3%, 95% CI 2.94%–6.06%]) than nonpedigree cats (39/1,300 [3%, 95% CI = 2.14%–4.08%]) were seropositive; however, this finding was not significant (p = 0.1). Purebred cats are more likely to be kept indoors only and may therefore experience more close contact with their owners, meaning they are more prone to exposure to SARS-CoV-2 if their owners become infected.

Although a definitive protective threshold antibody level for SARS-CoV-2 has not yet been established, waning neutralizing antibody levels in humans after vaccination have been associated with reinfection and reduced protection against novel variants (*11*). Sequential samples >12 days apart were collected from 5 seropositive cats. In all 5 cases, the neutralizing titers against SARS-CoV-2 waned over time. The average percentage decrease in titer per day was highly variable across samples, although for 3 of 5 cats it was consistent across all variants (Table).

**Table:** Overview of longitudinal samples used in study of SARS-CoV-2 seroprevalence and immunity in cats, United Kingdom, April 2020–February 2022*

Sample	Days between sampling	Titer				% Decrease per day		
		B.1	Alpha	Delta		B.1	Alpha	Delta
Cat F	12	490	257	601		5.90	0.90	4.10
		146	229	303				
Cat G	175	586	677	243		0.40	0.40	0.40
		134	170	58				
Cat H	94	687	825	2,165		0.30	0.20	0.70
		474	678	685				
Cat J	175	627	719	247		0.30	0.40	0.40
		318	241	79				
Cat L	23	109	102	468		−7.20	1.40	1.60
		289	70	301				

*We used 2 samples from each of 5 animals, taken >12 d apart. The earlier sample was used in the overall analysis; however, newer samples were also tested. Values related to each variant are shown for each sample, with the earlier sample above and later below. Titers are color-coded by size (stronger titers and greater decreases are shown with darker shading). B.1 indicates ancestral/wild-type virus.

## Conclusions

This study demonstrated increasing seroprevalence of SARS-CoV-2 antibodies in the UK domestic cat population, consistent with results reported in a survey of cats and dogs recently conducted in Canada (*12*) and the low seroprevalence observed during the first and second waves of the pandemic (*13*,*14*). This increase could be explained by the persistence of the humoral response over time, with a consequent accumulation in the number of seropositive results in the population. In addition, increased seroprevalence during the later months of the pandemic may mean the likelihood of human-to-cat transmission is greater for newer variants that have previously been shown to be more readily transmitted between humans (*15*), although this hypothesis has not been confirmed experimentally.

This study demonstrates the importance of adopting a One Health approach to monitor SARS-CoV-2 infections in pet cats that are in close contact with their SARS-CoV-2–positive owners. Changes in transmissibility of emerging variants should be monitored in cats as well as humans.

This article was preprinted at https://www.biorxiv.org/content/10.1101/2022.11.18.517046v1.

Appendix 1 Additional information about seropositivity and cross-variant antibody neutralization in cats, United Kingdom.

Appendix 2 Samples used in study of seropositivity and cross-variant antibody neutralization in cats, United Kingdom.
