# Supplementary material for: SARS-CoV-2 Seroprevalence and Cross-Variant Antibody Neutralization in Cats, United Kingdom
Source: Emerg Infect Dis. 2023 Jun;29(6):1223–7. doi: 10.3201/eid2906.221755 (PMC10202862; doi:10.3201/eid2906.221755)
Supplement: Appendix 1 — Additional information about seropositivity and cross-variant antibody neutralization in cats, United Kingdom. [file 22-1755-Techapp-s1.pdf]

Article DOI: https://doi.org/10.3201/eid2906.221755

SARS-CoV-2 Seroprevalence and Cross-Variant Antibody Neutralization in Cats, United Kingdom

Appendix 1

Methods

Samples

Residual blood samples for serologic testing were obtained from the University of Glasgow Veterinary Diagnostic Services laboratory (VDS). These samples had been submitted by practicing UK veterinary surgeons for purposes including routine monitoring, pre-breeding screening, testing for other infections and the diagnosis of hormonal disorders. Residual serum/plasma that would otherwise have been discarded after all requested tests had been completed was used for this study. None of the samples had been submitted because of suspected SARS-CoV-2 infection. These samples represented a cohort broadly representative of the domestic cat population throughout the UK. Poor quality samples, for example those displaying marked hemolysis, were excluded. Ethical approval for the study and was granted by the University of Glasgow Veterinary Ethics Committee (EA27/20). Samples were given a unique identification number on arrival, and investigators (GT, NL and SJ) were blinded to sample metadata until the data analysis stage.

Serologic Testing

Samples were initially screened at a final dilution of 1 in 100 using a pseudotype-based virus neutralization assay (PVNA). PVNA positive samples were confirmed using a double antigen binding assay (DABA) ELISA that detected antibodies recognizing the receptor-binding domain of the SARS-CoV-2 S protein. Neutralizing antibody titers were estimated by performing a PVNA with serially diluted samples.

For the neutralization assays, HIV (SARS-CoV-2) pseudotypes were constructed bearing the spike proteins of either the Wuhan-Hu-1 D614G (B.1), Alpha (B.1.1.7), Delta (B.1.617.2) or

Omicron (BA.1) SARS-CoV-2 variants. Samples collected early in the pandemic were tested against Wuhan-Hu-1 D614G (B.1) only while new variants were included in the assay over time, as each new SARS-CoV-2 variant emerged during subsequent waves of the pandemic.

Pseudotype-Based Virus Neutralization Assay

The method for this assay has been described previously (*1*). Briefly, HEK293, HEK293T, and 293-ACE2 cells were maintained in Dulbecco's modified Eagle's medium (DMEM) supplemented with 10% fetal bovine serum, 200mM L-glutamine, 100μg/ml streptomycin and 100 IU/ml penicillin. HEK293T cells were transfected with the appropriate SARS-CoV-2 S gene expression vector (wild type or variant) in conjunction with p8.91 (*2*) and pCSFLW (*3*) using polyethylenimine (PEI, Polysciences, Warrington, USA). HIV (SARS-CoV-2) pseudotypes were harvested from culture fluids 48 hours post-transfection, filtered at 0.45μm, aliquoted and frozen at −80°C before use. The SARS-CoV-2 spike glycoprotein expression constructs were synthesized by GenScript (Netherlands). Constructs bore the following mutations relative to the Wuhan-Hu-1 sequence (GenBank: MN908947):

- **B.1 (Wuhan-Hu-1 D614G)** – D614G
- **B.1.1.7 (Alpha)** – Δ69–70, Δ144, N501Y, A570D, D614G, P681H, T716I, S982A, D1118H
- **B.1.617.2 (Delta)** – T19R, G142D, Δ156–157, R158G, L452R, T478K, D614G, P681R, D950N
- **B.1.1.529 (Omicron BA.1) -** A67V, Δ69–70, T95I, G142D/Δ143–145, Δ211/L212I, ins214EPE, G339D, S371L, S373P, S375F, K417N, N440K, G446S, S477N, T478K, E484A, Q493R, G496S, Q498R, N501Y, Y505H, T547K, D614G, H655Y, N679K, P681H, N764K, D796Y, N856K, Q954H, N969K, L981F

All synthesized S genes were codon-optimized, incorporated the mutation K1255STOP to enhance surface expression, and were cloned into the pcDNA3.1(+) eukaryotic expression vector. 293-ACE2 target cells (*4*) were maintained in complete DMEM supplemented with 2μg/ml puromycin.

The fixed dilution screen was performed with serum/plasma diluted 1:50 in complete DMEM (in duplicate) for each pseudotype. Diluted samples were incubated with HIV (SARS-CoV-2) pseudotypes for 1 hour and plated onto 239-ACE2 target cells. After 48–72 hours, luciferase activity was quantified by the addition of Steadylite Plus chemiluminescence substrate and analysis on a Perkin Elmer EnSight multimode plate reader (PerkinElmer, Beaconsfield, UK). Samples which reduced the infectivity of the pseudotypes by at least 90% were classed as positive. For positive samples, neutralizing activity was then quantified by serial dilution. Each sample was serially diluted (in triplicate) from 1:50 to 1:36450 in complete DMEM before incubation with the respective viral pseudotype. Antibody titer was then estimated by interpolating the point at which infectivity had been reduced to 90% of the value for the no serum control samples.

Seropositive cats were categorized according to the pseudotype variant against which the highest neutralizing titer was obtained. For example, samples showing a higher titer against the Delta pseudotype compared to the other pseudotypes were categorized as "Delta dominant."

Double Antigen Bridging Assay ELISA

All samples that appeared positive on the initial fixed dilution PVNA were tested using a species agnostic double antigen bridging assay (Microimmune SARS-CoV-2 Double Antigen Bridging Assay (COVT016), Clin-Tech, Guildford, England) according to the manufacturer's instructions, to determine whether samples contained antibodies to the B.1 SARS-CoV-2 receptor-binding domain. This was used to confirm results of the pseudotype-based neutralization assay by confirming low chemiluminescence readings were caused by high levels of antibody rather than any toxic contamination of samples killing the cells.

Data Analysis

Duplicate samples were removed while samples from the same animal tested multiple times were identified and the earliest sample was used to estimate seroprevalence. A small number of animals had multiple samples submitted to the VDS at different times and, using these samples, longitudinal titers were tabulated to explore the effect of time on the development of the humoral response to SARS-CoV-2. Data were analyzed and graphs prepared using GraphPad Prism 9.3.1. and Microsoft Excel. Distribution of data was assessed using a Shapiro-Wilk Normality test. Sample metadata (age, sex, location, breed) was acquired from information

recorded in the VDS database, which was supplied by submitting veterinary surgeons Differences between groups were assessed for significance in paired data using a Wilcoxon test and in unpaired data using a Mann-Whitney test or ANOVA. Significance of categorical data was assessed using a Chi-Square test.

References

1. Davis C, Logan N, Tyson G, Orton R, Harvey WT, Perkins JS, et al.; COVID-19 Genomics UK (COG-UK) Consortium; COVID-19 DeplOyed VaccinE (DOVE) Cohort Study investigators. Reduced neutralisation of the Delta (B.1.617.2) SARS-CoV-2 variant of concern following vaccination. PLoS Pathog. 2021;17:e1010022. PubMed https://doi.org/10.1371/journal.ppat.1010022

2. Zufferey R, Nagy D, Mandel RJ, Naldini L, Trono D. Multiply attenuated lentiviral vector achieves efficient gene delivery in vivo. Nat Biotechnol. 1997;15:871–5. PubMed https://doi.org/10.1038/nbt0997-871

3. Zufferey R, Dull T, Mandel RJ, Bukovsky A, Quiroz D, Naldini L, et al. Self-inactivating lentivirus vector for safe and efficient in vivo gene delivery. J Virol. 1998;72:9873–80. PubMed https://doi.org/10.1128/JVI.72.12.9873-9880.1998

4. Hughes EC, Amat JAR, Haney J, Parr YA, Logan N, Palmateer N, et al. Severe acute respiratory syndrome coronavirus 2 serosurveillance in a patient population reveals differences in virus exposure and antibody-mediated immunity according to host demography and healthcare setting. J Infect Dis. 2021;223:971–80. PubMed https://doi.org/10.1093/infdis/jiaa788

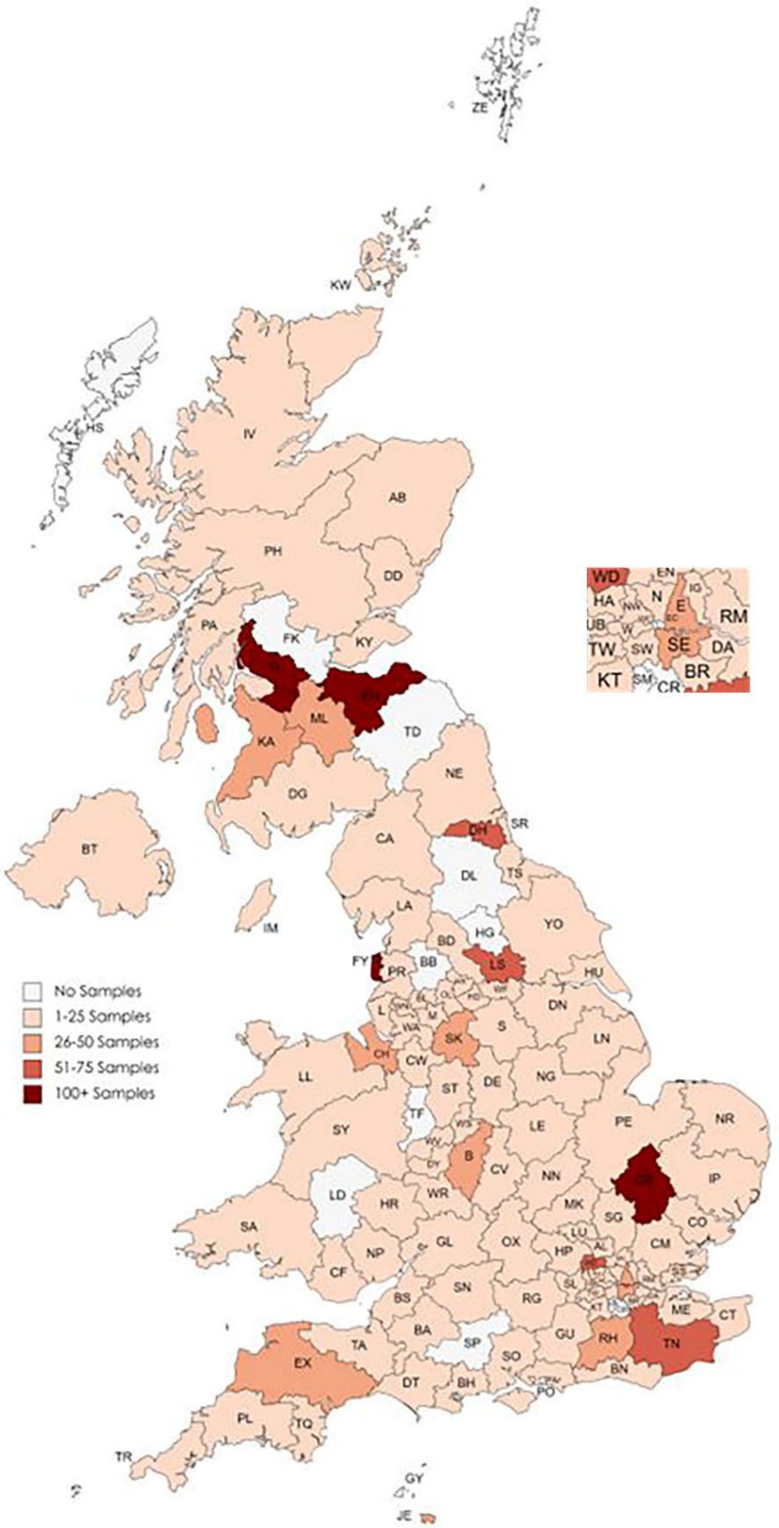

Appendix Figure 1. The location of the veterinary practices within the United Kingdom that submitted samples used in this study of seroprevalence in pet and stray cats.

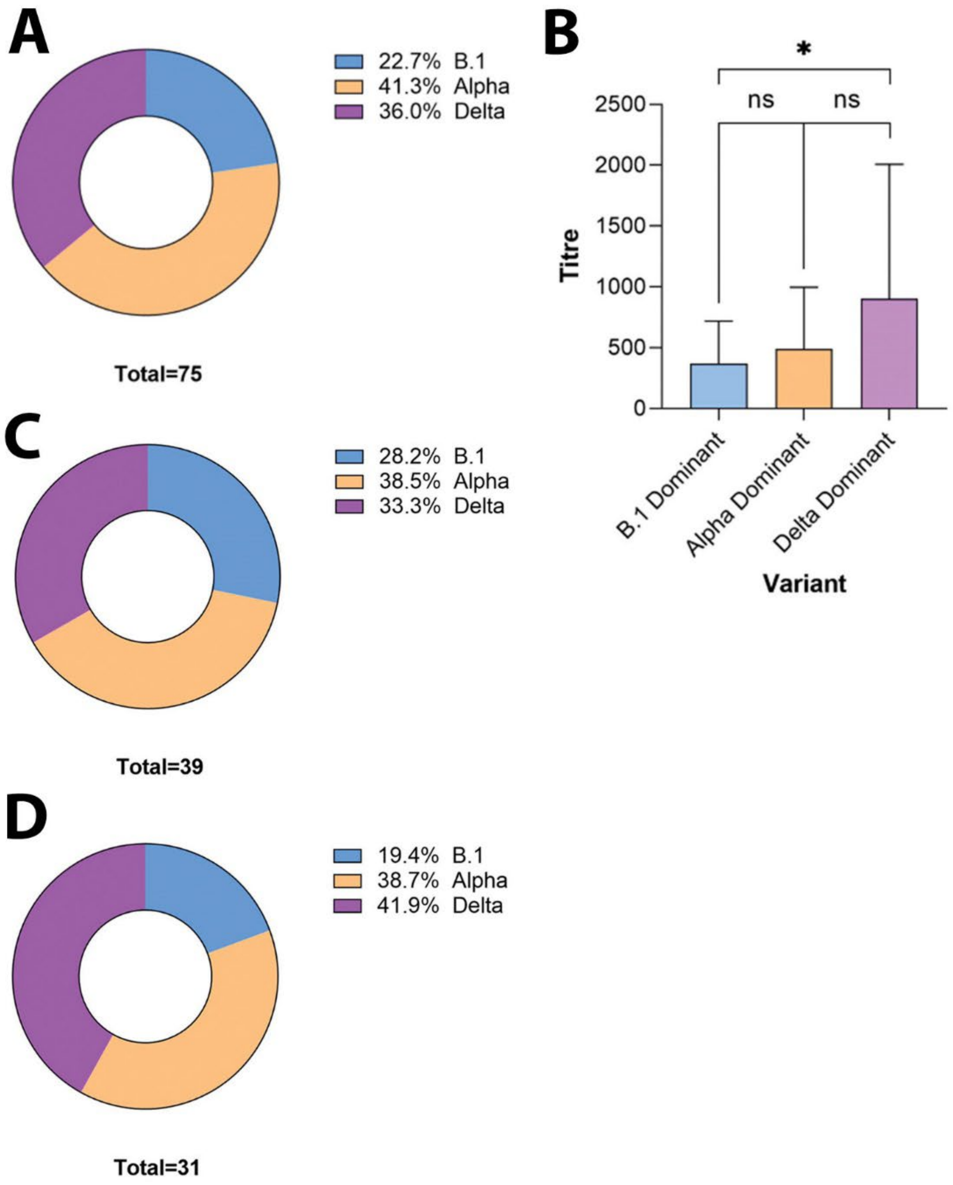

Appendix Figure 2. Seropositive cases shown by dominant variant. Seropositive samples categorized by their dominant variant (A). The average titer produced by each serum sample against its dominant variant (B). Normality of sample distribution was assessed using a Shapiro-Wilk test and significance was assessed using a Mann-Whitney test (ns, not significant; *, $p < 0.05$). Seropositive samples categorized by breed – either nonpedigree (C) or pedigree (D). Seropositive cats of unknown breed were not included in this figure.

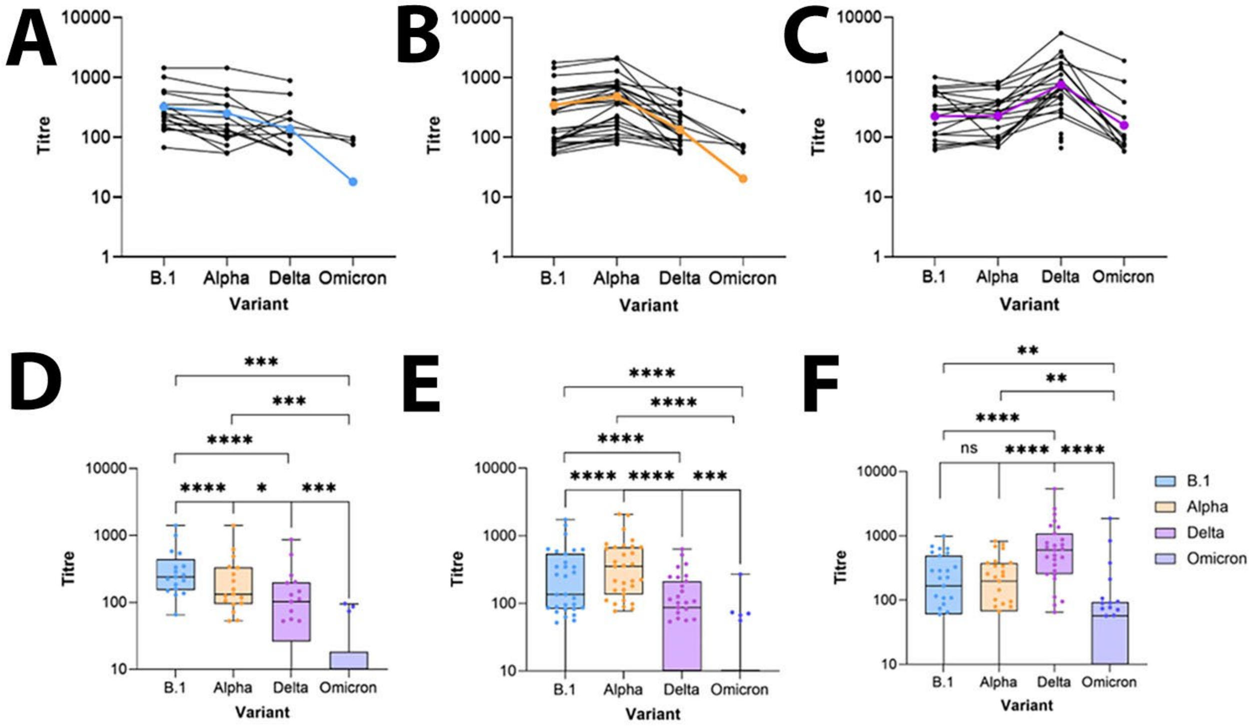

Appendix Figure 3. Virus neutralization titers of seropositive samples grouped by dominant variant. Neutralizing titers for samples classified by dominant variant, showing the 3 distinct patterns of immunity (ns, not significantly different. Asterisks indicate significant differences as follows: *, $p<0.05$; **, $p<0.01$; ***, $p<0.001$; ****, $p<0.0001$, by Wilcoxon test). Mean patterns of cross-neutralization for each dominant group are displayed in bold color in line graphs.
